# Perilipin Overexpression in White Adipose Tissue Induces a Brown Fat-Like Phenotype

**DOI:** 10.1371/journal.pone.0014006

**Published:** 2010-11-16

**Authors:** Takashi Sawada, Hideaki Miyoshi, Kohei Shimada, Akira Suzuki, Yuko Okamatsu-Ogura, James W. Perfield, Takuma Kondo, So Nagai, Chikara Shimizu, Narihito Yoshioka, Andrew S. Greenberg, Kazuhiro Kimura, Takao Koike

**Affiliations:** 1 Department of Internal Medicine II, Hokkaido University Graduate School of Medicine, Sapporo, Japan; 2 Department of Biomedical Sciences, Hokkaido University Graduate School of Veterinary Medicine, Sapporo, Japan; 3 Department of Pathology, KKR Sapporo Medical Center, Sapporo, Japan; 4 Department of Nutrition and Exercise Physiology and Food Science, University of Missouri, Columbia, Missouri, United States of America; 5 Jean Mayer United States Department of Agriculture Human Nutrition Research Center on Aging, Tufts University, Boston, Massachusetts, United States of America; University of Tor Vergata, Italy

## Abstract

**Background:**

Perilipin A (PeriA) exclusively locates on adipocyte lipid droplets and is essential for lipid storage and lipolysis. Previously, we reported that adipocyte specific overexpression of PeriA caused resistance to diet-induced obesity and resulted in improved insulin sensitivity. In order to better understand the biological basis for this observed phenotype, we performed additional studies in this transgenic mouse model.

**Methodology and Principal Findings:**

When compared to control animals, whole body energy expenditure was increased in the transgenic mice. Subsequently, we performed DNA microarray analysis and real-time PCR on white adipose tissue. Consistent with the metabolic chamber data, we observed increased expression of genes associated with fatty acid β-oxidation and heat production, and a decrease in the genes associated with lipid synthesis. Gene expression of *Pgc1a*, a regulator of fatty acid oxidation and *Ucp1*, a brown adipocyte specific protein, was increased in the white adipose tissue of the transgenic mice. This observation was subsequently verified by both Western blotting and histological examination. Expression of RIP140, a regulator of white adipocyte differentiation, and the lipid droplet protein FSP27 was decreased in the transgenic mice. Importantly, FSP27 has been shown to control gene expression of these crucial metabolic regulators. Overexpression of PeriA in 3T3-L1 adipocytes also reduced FSP27 expression and diminished lipid droplet size.

**Conclusions:**

These findings demonstrate that overexpression of PeriA in white adipocytes reduces lipid droplet size by decreasing FSP27 expression and thereby inducing a brown adipose tissue-like phenotype. Our data suggest that modulation of lipid droplet proteins in white adipocytes is a potential therapeutic strategy for the treatment of obesity and its related disorders.

## Introduction

The metabolic syndrome is an accumulation of risk factors of cerebrovascular and cardiovascular disease such as diabetes, dyslipidemia and hypertension [1]. Increased visceral fat and elevated lipolysis cause dysfunction of various organs and abnormal production of adipokines [2]. Therefore, when considering the pathophysiology of the metabolic syndrome, it is extremely important to understand the mechanisms of lipid storage and release (lipolysis) in adipocytes. Within adipocytes, triglyceride is predominately stored within lipid droplets that are surrounded by a phospholipid monolayer containing various lipid droplet proteins. These proteins belong to the PAT family which contains Perilipin, ADRP/adipophilin, TIP47, MLDP (OXPAT/LSD5) and S3-12 which all have homology in their N-terminal sequence [3]. Perilipin (Peri) is the predominant protein present on the surface of lipid droplets in fat cells of white/brown adipose tissue and steroid producing cells [4]. Perilipin A (PeriA) is the most abundant adipocyte lipid phosphoprotein, which is activated by protein kinase A (PKA) and is considered to play a central role in regulating lipid metabolism in adipocytes by controlling various proteins [5]. Ablation of PeriA from white adipose tissue (WAT) causes dysregulation of adipocyte lipid storage characterized by increased basal lipolysis and decreased PKA-stimulated lipolysis and results in a dramatic reduction in WAT mass [6], [7]. The role of PeriA in WAT is to suppress lipolysis in the absence of PKA stimulation, and enhance lipolysis (∼100 fold) with PKA stimulation [8], [9].

Recently, fat specific protein 27 (FSP27 or Cidec) was identified as a protein which localizes on the surface of lipid droplets in white adipocytes and contributes to energy storage by promoting the formation of unilocular lipid droplets [10], [11]. FSP27 deficiency dramatically reduced WAT mass and induced a brown adipocyte-like morphology in the WAT via reducing the factors inhibiting brown adipocyte differentiation such as receptor interacting protein 140 (RIP140) and increasing brown adipocyte-specific genes or key metabolic controlling factors such as PPAR coactivator 1α (PGC1α[1112].

Previously, we generated transgenic mice which overexpressed either human or mouse PeriA specifically in adipocytes and studied these mice in the context of obesity and lipid/glucose metabolism [13]. When challenged with a high fat diet (HFD), both human and mouse PeriA Tg mice gained less weight, and had reduced WAT mass, though their food intake was similar to that of wild type (WT) mice. In this manuscript, we performed further studies in this human PeriA Tg mouse model to investigate the mechanisms of obesity-resistance and metabolic changes.

## Results

### Increased oxygen consumption and energy expenditure in PeriA Tg mice

**Figure 1:**
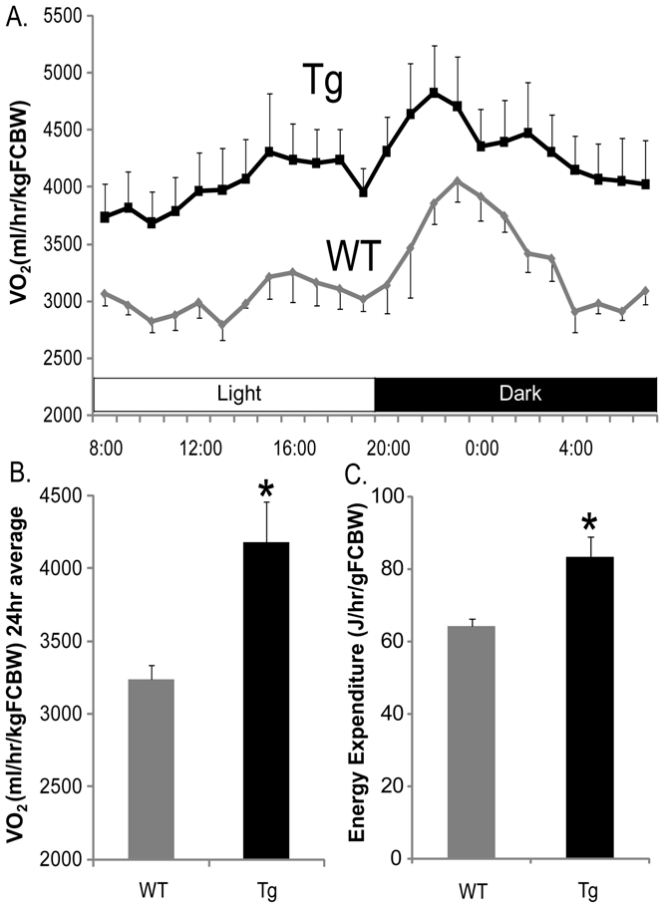
Oxygen consumption and energy expenditure. (A) Whole-body oxygen consumption rate during a 12-hour dark/12-hour light cycle for 30-week-old mice fed a high fat diet (n = 7 for WT; n = 7 for Tg). (B) The average value during the 24-hour period. (C) Energy expenditure during 24-hour period in the experiment. FCBW: fat-corrected body weight = body weight−(mass of subcutaneous and perigonadal white adipose tissue). Data are mean ± SEM. *, *p*<0.05.

Consistent with our previous study [13], body weight and subcutaneous and gonadal WAT mass were reduced in HFD-fed Tg mice as compared to HFD-fed WT mice (data not shown). With regard to energy metabolism, whole-body oxygen consumption rate (VO_2_) in HFD-fed human PeriA Tg mice was markedly higher than that of WT controls (Figure 1A). Twenty-four-hour oxygen consumption and energy expenditure were significantly increased in Tg mice maintained on a HFD and this difference was maintained after correction for differences in fat pad mass compared with the corresponding values for WT mice (Figure 1B, C). Furthermore, we measured oxygen consumption of white adipocytes isolated from PeriA Tg and wild-type mice to examine their mitochondrial function. Consistent with our metabolic chamber data *in vivo*, both basal and norepinephrine (NE) stimulated oxygen consumption tended to be increased in white adipocytes isolated from PeriA Tg mice (Figure S1).

### Up-regulation of brown fat associated transcriptional factors, and down-regulation of lipid synthesis genes in WAT of Tg mice

**Figure 2:**
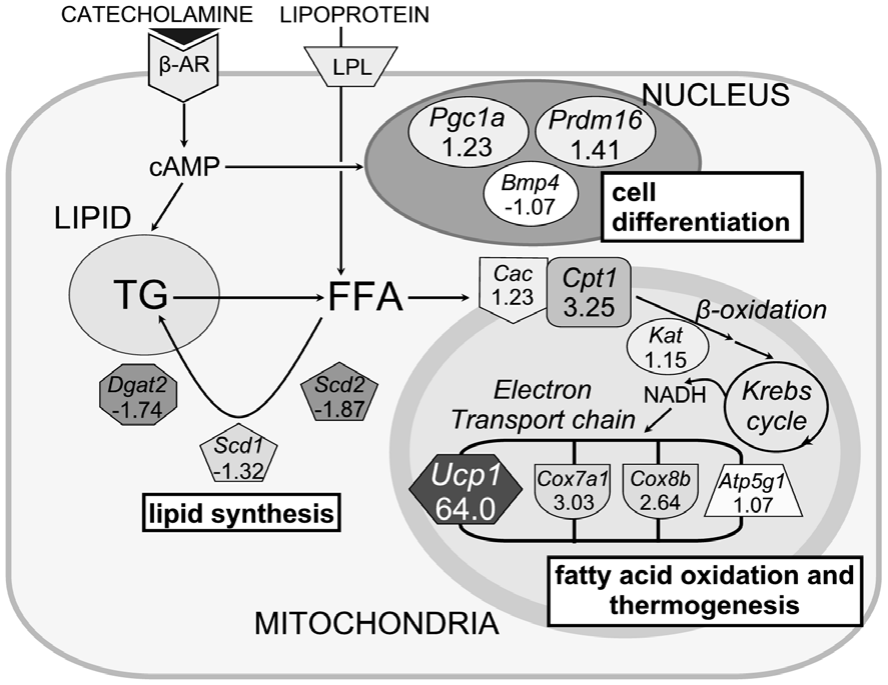
DNA microarray analysis. Metabolic pathways altered in WAT of Tg mice: cell differentiation, lipid synthesis, fatty acid oxidation, and thermogenesis. Fold changes in transcript levels are noted beneath the gene symbols. *Atp5g1*: ATP synthase, H+ transporting, F1 gamma 1, *Bmp*: bone morphogenic protein, *Cac*: carnitine/acylcarnitine translocase, *Cox*: cytochrome c oxidase, *Cpt*: carnitine palmitoyl transferase, *Dgat*: diacylglycerol acyltransferase, *Kat*: 3-ketoacyl-CoA thiolase, *Pgc1a*: peroxisome proliferator activated receptor gamma coactivator-1 alpha, *Prdm16*: PRD1-BF1-RIZ1 homologous domain containing 16, *Scd*: stearoyl-CoA desaturase.

**Figure 3:**
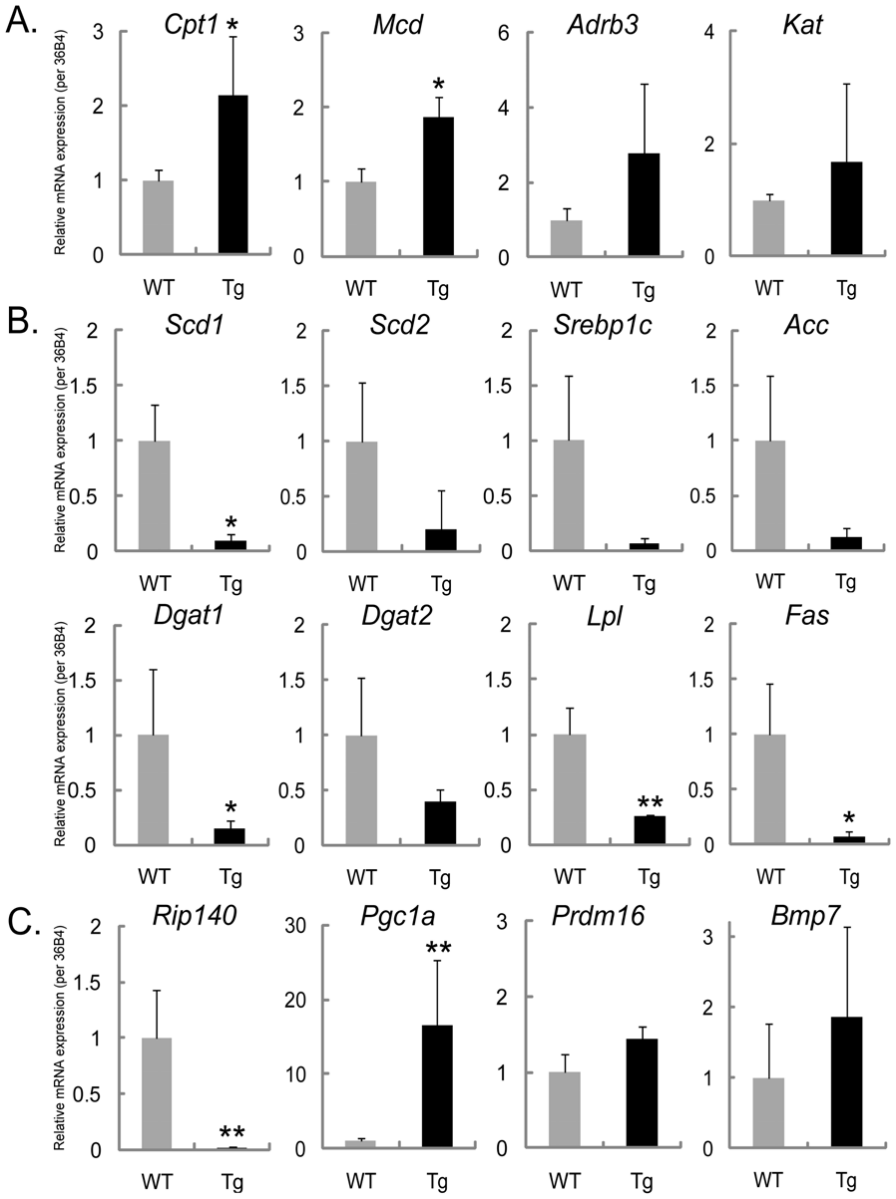
Quantitative PCR. Quantitative real-time PCR analysis of the expression of genes in WAT of 30-week-old WT and Tg mice fed a high fat diet, related to (A) fatty acid oxidation and thermogenesis, (B) lipid synthesis, and (C) cell differentiation to white/brown adipocytes. Data were normalized by the amount of *36B4* mRNA and expressed relative to the corresponding value for WAT of WT mice; Data are mean ± SEM (n = 9). *, *p*<0.05; **, *p*<0.01. *Acc*: acetyl-CoA carboxylase, *Adrb3*: beta-3 adrenergic receptor, *Fas*: fatty acid synthease, *Lpl*: lipoprotein lipase, *Mcd*: malonyl-CoA decarboxylase, *Srebp1c*: sterol regulatory element binding protein 1c.

Subsequently we employed DNA microarray analysis on WAT from Tg and WT mice. We observed increased expression of genes associated with fatty acid β-oxidation and heat production, and a decrease in the genes involved in lipid synthesis (Table 1, Figure 2). To validate these microarray data, we next performed real-time PCR on selected gene targets. Consistent with the metabolic chamber data, we confirmed significant increases in the expression of *Cpt1* (WT 1.00±0.15 *vs.* Tg 2.13±0.81, *p* = 0.049) and *Mcd* (WT 1.00±0.18 *vs.* Tg 1.87±0.26, *p* = 0.021) that are involved in fatty acid β-oxidation and energy expenditure (Figure 3A). On the other hand, we observed significant decreases in the expression of the lipogenic genes *Scd1* (WT 1.00±0.33 vs. Tg 0.10±0.06, *p* = 0.014), *Dgat1* (WT 1.00±0.59 vs. Tg 0.15±0.07, *p* = 0.018), *Lpl* (WT 1.00±0.24 vs. Tg 0.25±0.02, *p* = 0.002) and *Fas* (WT 1.00±0.46 vs. Tg 0.08±0.04, *p* = 0.016). Similarly, the expression of *Scd2*, *Dgat2*, *Srebp1c* and *Acc* tended to be decreased (Figure 3B). Notably, expression of *Rip140* which is involved in the differentiation of white adipocytes was remarkably decreased (WT 1.00±0.43 *vs.* Tg 0.02±0.01, *p* = 0.002), while *Pgc1α*, a regulator of mitochondrial biogenesis, was significantly increased (WT 1.00±0.42 *vs.* Tg 16.59±8.66, *p* = 0.003) (Figure 3C).

**Table 1:** Gene expression of WAT in microarray analysis.

Gene/protein name (symbol)	UniGene ID	Fold change
**Adipokines**		
Adiponectin receptor 2 (Adipor2)	Mm.291826	1.52
Adiponectin receptor 1 (Adipor1)	Mm.259976	1.15
Adiponectin (Adipoq)	Mm.3969	1.00
Lipoprotein lipase (Lpl)	Mm.1514	−1.07
Monocyte chemotactic protein-1; chemokine (C-C motif) ligand 2 (Ccl2)	Mm.290320	−1.23
Leptin (Lep)	Mm.277072	−1.52
**Adipocyte differentiation**		
PRD1-BF1-RIZ1 homologous domain containing 16 (Prdm16)	Mm.257785	1.41
Peroxisome proliferator activated receptor gamma, coactivator 1 alpha (Pgargc1a)	Mm.259072	1.23
**Fatty acid oxidation and thermogenesis**		
Uncoupling protein 1 (Ucp1)	Mm.4177	64.00
Carnitine parmitoyltransferase 1 (Cpt1)	Mm.34881	3.25
Carnitine/acylcarnitine translocase (Cac)	Mm.29666	1.23
3-Ketoacyl-CoA thiolase B (Kat)	Mm.30417	1.15
**Lipid synthesis**		
Stearoyl-CoA desaturase 1 (Scd1)	Mm.267377	−1.32
Diacylglycerol acyltransferase 2 (Dgat2)	Mm.180189	−1.74
Stearoyl-CoA desaturase 2 (Scd2)	Mm.193096	−1.87
**Electron transport chain**		
cytochrome c oxidase, subunit VIIa 1 (Cox7a1)	Mm.12907	3.03
cytochrome c oxidase, subunit VIIIb (Cox8b)	Mm.3841	2.64
ATP synthase, H+ transporting, F1 gamma 1 (Atp5g1)	Mm.258	1.07

### Ectopic expression of UCP1, a brown adipose tissue specific protein, in white adipose tissue of PeriA Tg mice

**Figure 4:**
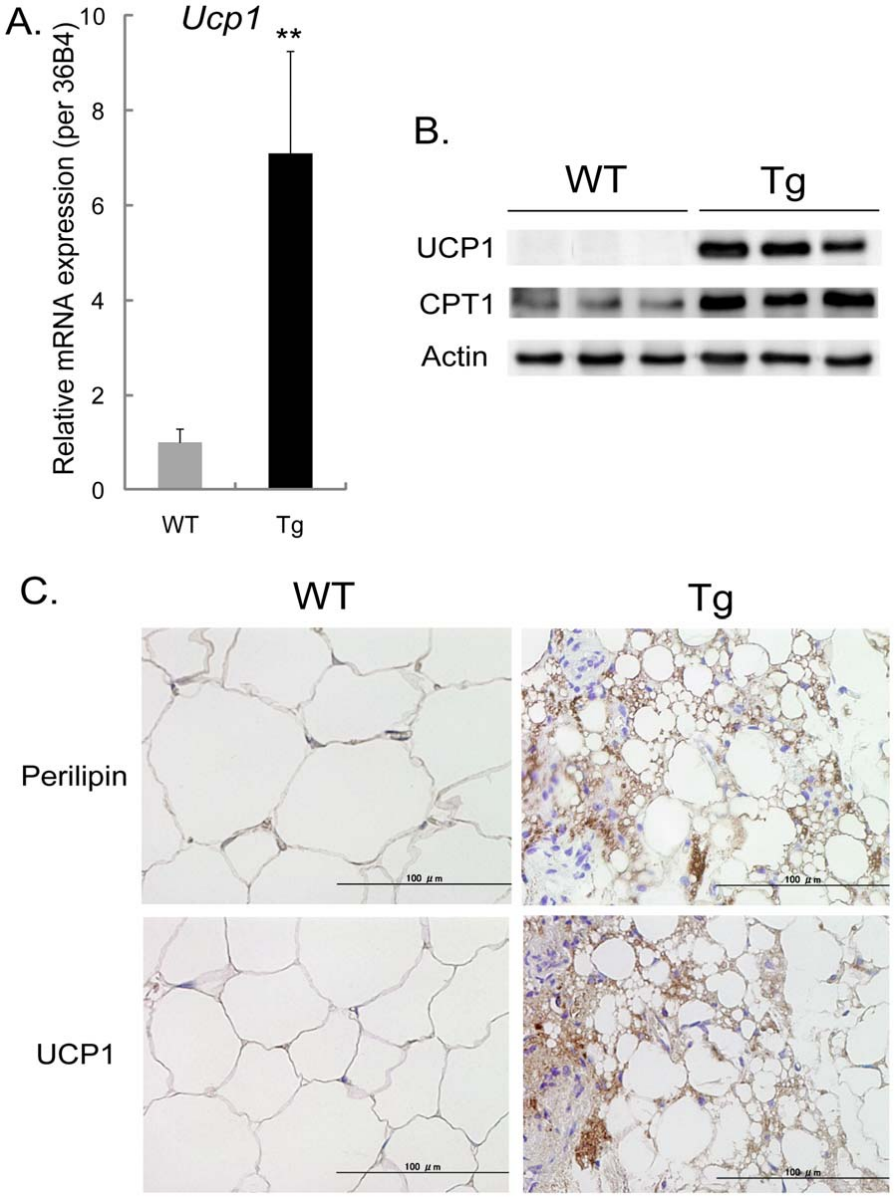
Ectopic expression of UCP1. Ectopic expression of UCP1 in WAT of 30-week-old PeriATg mice fed a chow diet. (A) Quantitative real-time PCR analysis of *Ucp1* mRNA expression in WAT of WT and Tg mice. Data are mean ± SEM (n = 7). **, *p*<0.01. (B) Western blot analysis showing UCP1 or CPT1 protein expression in WT and Tg mice. (C) Innunohistochemistry of PeriA or UCP1 in WAT of WT and Tg mice. Original magnification, ×400.

Uncoupling protein-1 (UCP1) functions to uncouple oxidative phosphorylation and converts the proton gradient energy to heat to maintain body temperature. It is thought to be expressed exclusively in brown adipocytes, and is considered a specific marker of brown adipocytes [14]. We confirmed the ectopic expression of *Ucp1* in the WAT of Tg mice by microarray and real-time PCR (Table 1, Figure 4A). UCP1 protein expression in the WAT of Tg mice was subsequently verified by both western blotting (Figure 4B) and histological immunostaining (Figure 4C). However, despite being increased, the UCP1 expression in WAT of Tg mice was much less than was present in brown adipose tissue (Figure S2). Histological analysis revealed that UCP1 was expressed in the smaller adipocytes in the WAT of Tg mice, but not in the WAT of WT mice. In addition, the previously reported increase in CPT1 gene expression (Figure 3A) in WAT of Tg mice was also confirmed by Western blotting (Figure 4B).

### The abundance of the lipid droplet protein FSP27 is reduced in the WAT of PeriA Tg mice

**Figure 5:**
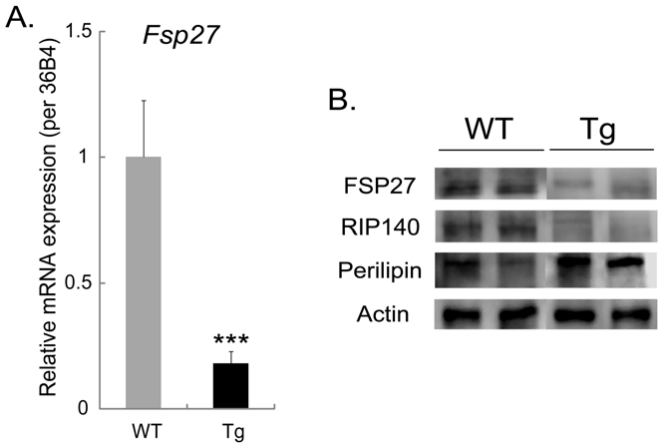
FSP27 expression *in vivo*. Alteration in the expression of lipid droplet proteins *in vivo*. (A) Quantitative real-time PCR analysis of *Fsp27* mRNA expression in WAT of WT and Tg mice. Data are mean ± SEM (n = 7). ***, *p*<0.001. (B) Western blot analysis for FSP27, RIP140 and PeriA in WAT of WT and Tg mice.

The mRNA expression of the lipid droplet protein *Fsp27* was significantly decreased in the WAT of PeriA Tg mice compared with WT mice (WT 1.00±0.22 *vs.* Tg 0.18±0.05, *p* = 0.0007) (Figure 5A). Similarly, protein expression of FSP27 and RIP140 were decreased in Tg mice (Figure 5B). As would be expected, the protein content of PeriA was clearly increased in the WAT of Tg mice when compared to control (Figure 5B).

### Expression of FSP27 was directly attenuated by PeriA overexpression *in vitro*

**Figure 6:**
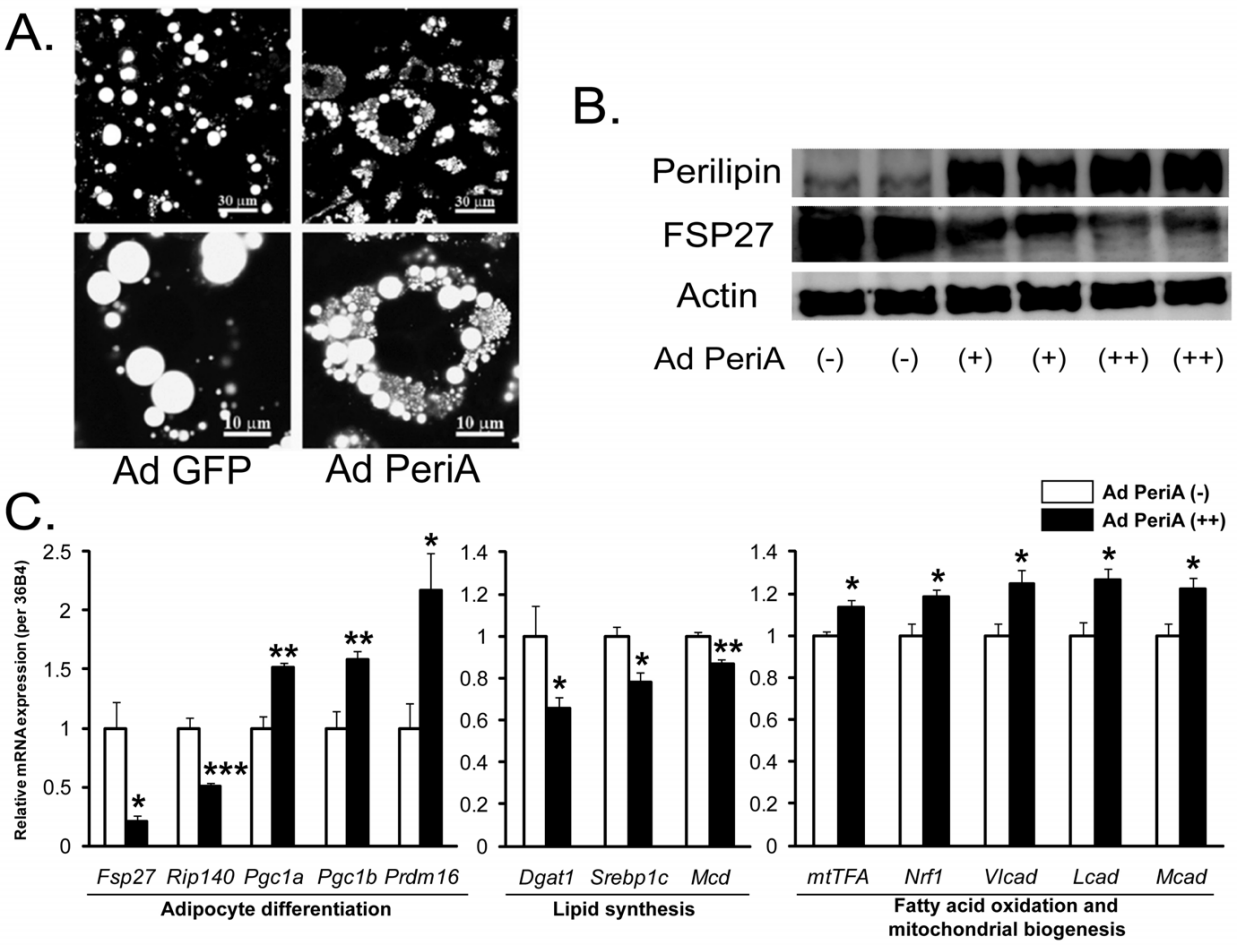
*In vitr*o experiments. Change of lipid droplet size and protein expression *in vitro*. (A) Microscopic images of 3T3-L1 adipocytes transfected human PeriA or GFP (control) using adenoviral system plus lipofection methods. (B) Western blot analysis for lipid droplet surface proteins (PeriA and FSP27) and Actin (as an internal control) in 30µg of lysates of day10 3T3-L1 cells treated with adenovirus PeriA (Ad PeriA). PeriA protein content increased dose-dependently with increasing PeriA viral titer in 3T3-L1 cells. Signs in a figure ((−), (+) and (++)) mean the amount of transfected adenovirus (none, single and double quantity). (C) Quantitative real-time PCR analysis of mRNA expression in cultured 3T3-L1 cells (white bar, Ad PeriA(−): black bar, Ad PeriA(++); Data are mean ± SEM (n = 6). *, *p*<0.05; **, *p*<0.01; ***, *p*<0.001). *mtTFA*: mitochondrial transcription factor A, *Nrf1*: nuclear respiratory factor 1, *Vlcad*: very long-chain acyl-CoA dehydrogenase, *Lcad*: long-chain acyl-CoA dehydrogenase, *Mcad*: medium-chain acyl-CoA dehydrogenase.

To further study the relationship between the expression of PeriA and FSP27 in adipocytes, we used an adenoviral system to overexpress human PeriA in cultured 3T3-L1 adipocytes. Overexpression of PeriA resulted in a dramatic reduction in lipid droplet size in cultured 3T3-L1 adipocytes (Figure 6A). This reduction in lipid droplet size was accompanied by a decrease in FSP27 protein expression (Figure 6B). These observations are consistent with our *in vivo* data from the WAT of the PeriA Tg mice. FSP27 locates on the surface of lipid droplets much like PeriA and functions to promote the formation of unilocular lipid droplet in white adipocytes [11]. Interestingly, our western blot data reveal that there is an inverse relationship between expression of PeriA and FSP27 (Figure 6B). Furthermore, we analyzed the mRNA expression level for genes involved in adipocyte differentiation, lipid synthesis, and fatty acid β-oxidation in PeriA-overexpressed 3T3-L1 samples. Consistent with our observations in the WAT of Tg mice, we observed down-regulation of *Rip140* and up-regulation of *Pgc1a*. Also, we observed increased expression of genes associated with fatty acid oxidation and mitochondrial biogenesis, and a decrease in the genes associated with lipid synthesis (Figure 6C).

## Discussion

It has been previously reported that Peri knockout mice lack the ability to store lipid due to a chronic low level of lipolysis, and therefore have diminished adipose depots and are obesity-resistant [6], [7]. We initially hypothesized that overexpression of PeriA in Tg mice would cause increased storage of lipid in adipose tissue and promote an obese phenotype. However, our original characterization of the PeriA Tg mice revealed that these animals had less adipose tissue and were resistant to diet-induced obesity [13]. We suggested that one mechanism underlying the resistance to HFD-induced obesity in PeriA Tg mice was an upregulation of oxidative genes in brown adipose tissue (BAT) [13]. In the current study, we further analyzed various metabolism-related proteins in WAT, and confirmed that the expression of genes related to β-oxidation and thermogenesis were increased in WAT as well as BAT. We performed a microarray to expand these initial findings. Our DNA microarray and RT-PCR data revealed an upregulation of *Ucp1*, a brown adipocyte specific protein, in WAT of Tg mice. This increase was also confirmed by Western blot analysis. Histological analysis of the WAT demonstrated the robust expression of UCP1 protein within small adipocytes that were located between larger white adipocytes. These small adipocytes are not observed in the WAT of WT mice and appear to have a morphology more similar to brown adipocytes. While only a small number of adipocytes appear to adopt this brown-like phenotype within the WAT of Tg mice, it has recently been reported that these “brite” (brown-in-white) adipocytes are metabolically significant as they can counteract obesity by contributing to thermogenesis [15]. The observed reduction in lipid droplet size and decreased mRNA expression of lipogenic genes in WAT of Tg mice might be a secondary change caused by increased fatty acid oxidation. However, it is possible that overexpression of PeriA results in a direct reduction in lipid droplet size and expression of lipogenic genes as we observed a reduction in lipid droplet size when PeriA was overexpressed in culture.

In recent reports about development and differentiation of adipocytes, it is recognized that the adipoblasts, which are derived from mesenchymal stem cells and exist around capillaries in adipose tissue, will be differentiated to brown adipocytes by factors such as PRDM16, BMP7, or PGC1α [16]–[19]. In contrast, when RIP140, a co-repressor of many nuclear receptors is present, the adipoblasts will be differentiated into white adipocytes [16], [20]. RIP140 is also reported to interact directly with PGC1α and suppress its activity [21]. These facts suggest the possibility that progenitor adipocytes could develop into brown adipocytes by changing the gene expression of these key metabolic controlling factors. In this study we observed a remarkable decrease of *Rip140* mRNA and increase of *Pgc1a* mRNA. These data suggest a potential mechanism for the observed increase in UCP1 expression with PeriA overexperssion. We hypothesize that PeriA overexpression alters the expression of these key regulators of differentiation in adipoblasts which are present in WAT and causes these progenitor adipocytes to differentiate into brown adipocytes.

Overexpression of PeriA *in vivo* causes a reduction in FSP27 protein expression. Consistent with this observation, we demonstrated *in vitro* an inverse relationship exists between FSP27 protein expression and PeriA expression. This phenomenon is likely due to a simple competition theory because both proteins preferentially locate to lipid droplets. Recently, FSP27 knockout mice have been described to have a phenotype of obesity-resistance, elevated oxygen consumption, extremely reduced WAT mass and smaller white adipocytes with multilocular lipid droplets [11], [12]. In addition, genes related to fatty acid β-oxidation and mitochondrial biosynthesis were significantly increased in FSP27 knockout mice [11], [12]. These data are very similar to the data presented in this manuscript where we report that PeriA overexpression causes a significant reduction in FSP27 which is associated with decreased adipocyte size, upregulation of genes involved in fatty acid oxidation and a decrease in lipogenic genes. Our data suggest that the attenuation of FSP27 by overexpression of PeriA was the trigger for the down-regulation of RIP140 and up-regulation of PGC1α, which caused progenitor adipocytes to differentiate into brown adipocytes rather than white adipocytes. This increase in brown adipocyte-like metabolic activity resulted in an increase in energy expenditure. Although FSP27 acts as a regulator to control gene expression of crucial metabolic regulators [12], the mechanism of how the lipid droplet related structural protein FSP27 reduces the expression of transcriptional factor RIP140 has not been clarified and requires further investigation. We hypothesize that a change of intracellular environment in the adipocyte itself such as “the size of the lipid droplets” or “the composition of the lipid droplet proteins”, can alter the characteristics of the adipocyte.

We revealed that PeriA overexpression results in resistance to diet-induced obesity, increased energy expenditure and reduced lipid synthesis *in vivo*, and that the basis for these effects was the induction of a a BAT-like phenotype in WAT due to a decrease in FSP27 expression. Our data suggest that modulation of lipid droplet proteins in white adipocytes is a potential therapeutic strategy for the treatment of obesity and its related disorders.

## Materials and Methods

### Ethics Statement

All animal care and experimental procedures in this study were approved by Hokkaido University Animal Experiment Committee (approval number: 08-0358) and carried out according to guidelines for animal experimentation of Hokkaido University.

### Antibodies

Polyclonal anti-perilipin antibody was generated as previously described [22]. Anti-UCP1 antiboby was a kind gift from Prof. Teruo Kawada (University of Kyoto, Kyoto, Japan) [23], and anti-FSP27 antibody was generated by Dr. Yoshikazu Tamori, et al (Kobe University, Kobe, Japan) [11]. Anti-RIP140 antibody was purchased from Abcam plc (Cambridge, UK). Anti-actin antibody, anti-CPT1 antibody, and horseradish peroxidase-conjugated anti-goat IgG were purchased from Santa Cruz Biotechnology Inc (Santa Cruz, CA). Horseradish peroxidase-conjugated anti-rabbit IgG was purchased from BIO-RAD Laboratories (Hercules, CA).

### Animal experiments

We generated transgenic mice that overexpressed human PeriA using the adipocyte specific aP2 promoter/enhancer [13]. All PeriA Tg mice used for the study were female, and heterozygous for the transgene. Littermates that lacked the transgene were used as controls (WT). Relative to WT littermates, PeriA protein levels were increased 2-fold in WAT and 5-fold in BAT of Tg mice. Mice were housed in a pathogen-free barrier facility at the Institute for Animal Experimentation at Hokkaido University. All mice were housed at room temperature, maintained on a 12 h light/dark cycle, given free access to water, and fed a standard chow diet (5.3% calories from fat; Oriental Yeast Co., ltd. MF, Tokyo, Japan) or a HFD (60% calories from fat; Research Diets #D12492, New Brunswick, NJ) for 25 weeks (from the age of 5 weeks to 30 weeks). Food intake and body weight were monitored weekly. On the day prior to tissue harvest at 30 weeks, food was removed at 21:00 h for an overnight fast. After the anesthetization with isoflurane (Abbott Japan, Tokyo, Japan), WAT from visceral (perigonadal) and subcutaneous depots were rapidly dissected out and processed for subsequent analysis.

### Measurement of oxygen consumption of HFD-fed Tg mice

Oxygen consumption of 30-week-old mice in the fed condition was measured with an open-circuit-type metabolic chamber (MM202R; Muromachi Kikai, Tokyo, Japan) every 3 minutes for 48 hours (12-hour dark/12-hour light cycle) at 25°C. Data were normalized to fat-corrected body weight (BW – the mass of subcutaneous and perigonadal white adipose tissue). Energy expenditure was calculated using the following formula: ([1.07×respiratory quotient +3.98]× VO_2_ ×4.2×60/body weight of a mouse). Measurement of oxygen consumption in isolated white adipocytes was performed as previously described [11].

### DNA microarray analysis

Total RNA extraction was performed by using Ribopure Kit (Ambion, Austin, TX), according to the manufacturer's instructions. To minimize individual variation as a source of gene-expression variance, RNA samples were pooled, one pool representing the three HFD-fed Tg mice (4µg from each sample) and one representing the three HFD-fed WT mice (4µg from each sample). RNA (2µg from each pool) was reverse transcribed to cDNA and tagged with biotin with one-cycle target labeling and hybridized according to the standard protocol using Mouse Genome 430 2.0 array (Affymetrix), which was then washed, stained, and scanned. The results obtained from these samples were analyzed with the GeneChip Operating Software ver1.4. The detection algorithm uses probe pair (includes perfect-match (PM) and mismatch (MM)) intensities to generate a detection *p*-value and assign a Present, Marginal, or Absent call. A two-step procedure determines the detection *p*-value for a given probe set: the first step calculates the discrimination score R for each probe pair, by a formula R = (PM−MM)/(PM+MM). The second step tests the discrimination scores against the user-definable threshold *Tau*. The One-Sided Wilcoxon's Signed Rank test is the statistical method employed to generate the detection *p*-value. It assigns each probe pair a rank based on how far the probe pair discrimination score is from *Tau*. The detection *p*-value cut-offs, *Alpha 1* (*α1*) and *Alpha 2* (*α2*), provide boundaries for defining Present, Marginal, or Absent calls. Any *p*-value that falls below *α1* is assigned a ‘Present’ call, and above *α2* is assigned an ‘Absent’ call. Marginal calls are given to probe sets which have *p*-values between *α1* and *α2*. The settings of quantitation parameters were: *α1* = 0.05, *α2* = 0.065, *Tau* = 0.015. The genes detected to be ‘Present’ in the data from the microarray were passed to further analysis. Signal is a quantitative metric calculated for each probe set, which represents the relative level of expression of a transcript. The signal log ratio estimates the magnitude and direction of change of a transcript, which is calculated by log_2_ (Signal of Tg/Signal of WT). Differentially expressed probe sets were selected based on filtering by signal log ratio under −0.3 or over 0.3. The microarray data files have been submitted to the Gene Expression Omnibus (GEO) and the accession number is GSE21754.

### Quantitative PCR

For each gene that was determined to have expression differences in the DNA microarray, we evaluated actual differences by real-time PCR. cDNA was synthesized from 0.5µg of total RNA (High Capacity RNA-to-cDNA kit, Applied Biosystems, Warrington, UK). Real-time PCR was performed in triplicate on a 7500 Fast Real Time PCR system in 20µl total volume reactions using SYBR® Green PCR Master Mix (Applied Biosystems, Warrington, UK). Primers were designed using Primer Express. Data were analyzed by comparative critical threshold (Ct) method [24] and normalized to an endogenous control gene (*36B4*: acidic ribosomal phosphoprotein P0). Percent difference was calculated by 2^−ddCt^. The primers (sense and antisense, respectively) were as follows: *36B4*, 5′- GAG GAA TCA GAT GAG GAT ATG GGA-3′ and 5′- AAG CAG GCT GAC TTG GTT GC-3′; *Scd1*, 5′- GAG GCC TGT ACG GGA TCA TA-3′, 5′- CAG CCG AGC CTT GTA AGT TC-3′; *Scd2*, 5′- TGT CGC TGA GGT CTG AAG C-3′, 5′- TGT GGT GGT GGC TGA GTA AG-3′; *Dgat1*, 5′- ACG GAT CAT TGA GCG TCT CT-3′, 5′- TAG AAC TCG CGG TCT CCA A-3′; *Dgat2*, 5′- AGG CCC TAT TTG GCT ACG TT-3′, 5′- CAT CAG GTA CTC GCG AAG C-3′; *Srebp1c*, 5′- ATC TCC TAG AGC GAG CGT TG-3′, 5′- TAT TTA GCA ACT GCA GAT ATC CAA G-3′; *Lpl*, 5′- AGT AGA CTG GTT GTA TCG GG-3′, 5′- AGC GTC ATC AGG AGA AAG G-3′; *Acc*, 5′- AAC ATC CCC ACG CTA AAC AG-3′, 5′- CTG ACA AGG TGG CGT GAA G-3′; *Fas*, 5′- CCC TTG ATG AAG AGG GAT CA-3′, 5′- GAA CAA GGC GTT AGG GTT GA-3′; *Rip140*, 5′- ATG GGT GTT GTC CCT TCC TC-3′, 5′- AAC TGC TCG CTC TCT CGT TC-3′; *Pgc1a*, 5′- ATG TGT CGC CTT CTT GCT CT-3′, 5′- CAC GAC CTG TGT CGA GAA AA-3′; *Prdm16*, 5′- CAG CAC GGT GAA GCC ATT C-3′, 5′- GCG TGC ATC CGC TTG TG-3′; *Bmp7*, 5′- CCT GTC CAT CTT AGG GTT GC-3′, 5′- GCC TTG TAG GGG TAG GAG AAG-3′; *Cpt1*, 5′- CCA ATC ATC TGG GTG CTG G-3′, 5′- AAG AGA CCC CGT AGC CAT CA-3′; *Mcd*, 5′- TCC CTG GAT TCA CCA AGT GG-3′, 5′- TTC CTC CCA TGC TCC TTC C-3′; *Adrb3*, 5′-GCT GAC TTG GTA GTG GGA CTC-3′, 5′- TAG AAG GAG ACG GAG GAG GAG-3′; *Kat*, 5′- TGG CAC TCT CTG GGT TGT G-3′, 5′- GCA GGT TGT CAC GCT ACT CA-3′; *Ucp1*, 5′- GAT GGT GAA CCC GAC AAC TT-3′, 5′- CTG AAA CTC CGG CTG AGA AG-3′; *mtTFA*, 5′- AGT TCC CAC GCT GGT AGT GT-3′, 5′- GCG CAC ATC TCG ACC C-3′; *Nrf1*, 5′- CAG CAA CCC TGA TGG CAC CGT GTC G-3′, 5′- GGC CTC TGA TGC TTG CGT CGT CTG G-3′; *Vlcad*, 5′- GAA TGA CCC TGC CAA GAA CGA-3′, 5′- ATG CCC ACA ATC TCT GCC AAG-3′; *Lcad*, 5′- GGA CTC CGG TTC TGC TTC CA-3′, 5′- TGC AAT CGG GTA CTC CCA CA-3′; *Mcad*, 5′- CAA CAC TCG AAA GCG GCT CA-3′, 5′- ACT TGC GGG CAG TTG CTT G-3′; *Fsp27*, 5′- GCC CAG TTC CTT CCT TTC TG-3′, 5′- AAC ACT CTC TCG CAC ACC TC-3′.

### Immunoblot analysis

Frozen subcutaneous WAT was homogenized in a Tris-EDTA buffer containing 10mM Tris/HCl (pH7.4) and 1mM EDTA, centrifuged for 10 minutes at 4°C, 800g and collected middle layer [25]. Equal amounts (30µg) of proteins were separated on 10% SDS-polyacrylamide gel and transferred to a nitrocellulose membrane. Primary antibodies used were UCP1 (1∶3000), CPT1 (1∶1000), FSP27 (1∶500), RIP140 (1∶1000), PeriA (1∶2000), and Actin (1∶2000). Actin was used as a loading control. Secondary antibodies were horseradish peroxidase-conjugated anti-rabbit IgG (UCP1, FSP27, RIP140 and PeriA) or anti-goat IgG (CPT1 and Actin). Western blot analysis was performed using Amersham ECL Advance Western Blotting Detection Kit (GE Healthcare, Little Chalfont, UK) and detection was made using a CCD-camera system LAS-4000UVmini (Fujifilm, Tokyo, Japan).

### Immunohistochemistry

Subcutaneous WAT were dissected, fixed, embedded in paraffin, and sectioned. Sections were deparaffinized, treated with 1% H_2_O_2_ methanol, pre-incubated with 10% goat serum and exposed to primary antibodies: UCP1 (1∶1000), perilipin as positive controls (1∶100). Sections were then incubated with Biotin-conjugated anti-rabbit IgG, treated with peroxidase-conjugated streptavidin, and stained with DAB substrate kit (Nichirei Bioscience, Tokyo, Japan).

### Cell culture and transfection

3T3-L1 preadipocytes were grown in Dulbecco's modified Eagle's medium (DMEM) containing 10% fetal calf serum (FCS) and seeded on 12-well plates. After reaching confluence, cells were differentiated using 10µg/ml insulin, 0.5mM 3-isobutyl-1-methylxanthine, 1µM dexamethasone in DMEM containing 10% fetal bovine serum (FBS). Following 48 h of incubation, medium was replaced with DMEM only containing 10% FBS, and recombinant adenovirus expressing the construct of PeriA was transduced into the cultured cells with LipofectAMINE Plus™ (Invitrogen, Carlsbad, CA) which protocols were previously described [9], [26], [27]. The human PeriA vector was generated and verified as previously described [13], and various amounts of the vector was transfected to investigate relationships between expressional levels of PeriA and FSP27, and size of lipid droplets [28]. 5 days after transfection, cells were fixed for histology or harvested for immunoblot analysis or quantitative PCR as described above.

### Statistical analysis

Data were analyzed using two-sided Student's *t*-test and significance was set a *p*<0.05. Results are presented as mean values ± SEM.

## Supporting Information

Figure 1 Oxygen consumption of adipocytes isolated from inguinal WAT of wild-type and PeriA Tg mice. An arrow indicates the addition of 10μM norepinephrine (NE). Data are mean ± SEM of values from three independent experiments.
(177KB TIF)

Figure 2 Western blot analysis showing UCP1 protein expression in both BAT and inguinal WAT of WT and Tg mice fed a chow diet. Equal amount (30μg) of proteins was electrophoresed. Actin was used as a loading control.
(511KB TIF)
