# Correction: Perilipin Overexpression in White Adipose Tissue Induces a Brown Fat-Like Phenotype

**DOI:** 10.1371/annotation/45438cdc-913a-4009-b2c1-5c1bc437c926

**Published:** 2010-11-22

**Authors:** Takashi Sawada, Hideaki Miyoshi, Kohei Shimada, Akira Suzuki, Yuko Okamatsu-Ogura, James W. Perfield, Takuma Kondo, So Nagai, Chikara Shimizu, Narihito Yoshioka, Andrew S. Greenberg, Kazuhiro Kimura, Takao Koike

The following information was missing from the Funding section: Dr. Greenberg’s (ASG) work was supported by grants American Diabetes Association Grant: 7-08-RA-57; by the USDA, Agricultural Research Service under Contract no. 58-1950-7-707; and NIH RO1-DK0822574 and 1RC2ES018781.

