# Supplementary figures and images for: Perilipin Overexpression in White Adipose Tissue Induces a Brown Fat-Like Phenotype
Source: PLoS One. 2010 Nov 16;5(11):e14006. doi: 10.1371/journal.pone.0014006 (PMC2982838; doi:10.1371/journal.pone.0014006)

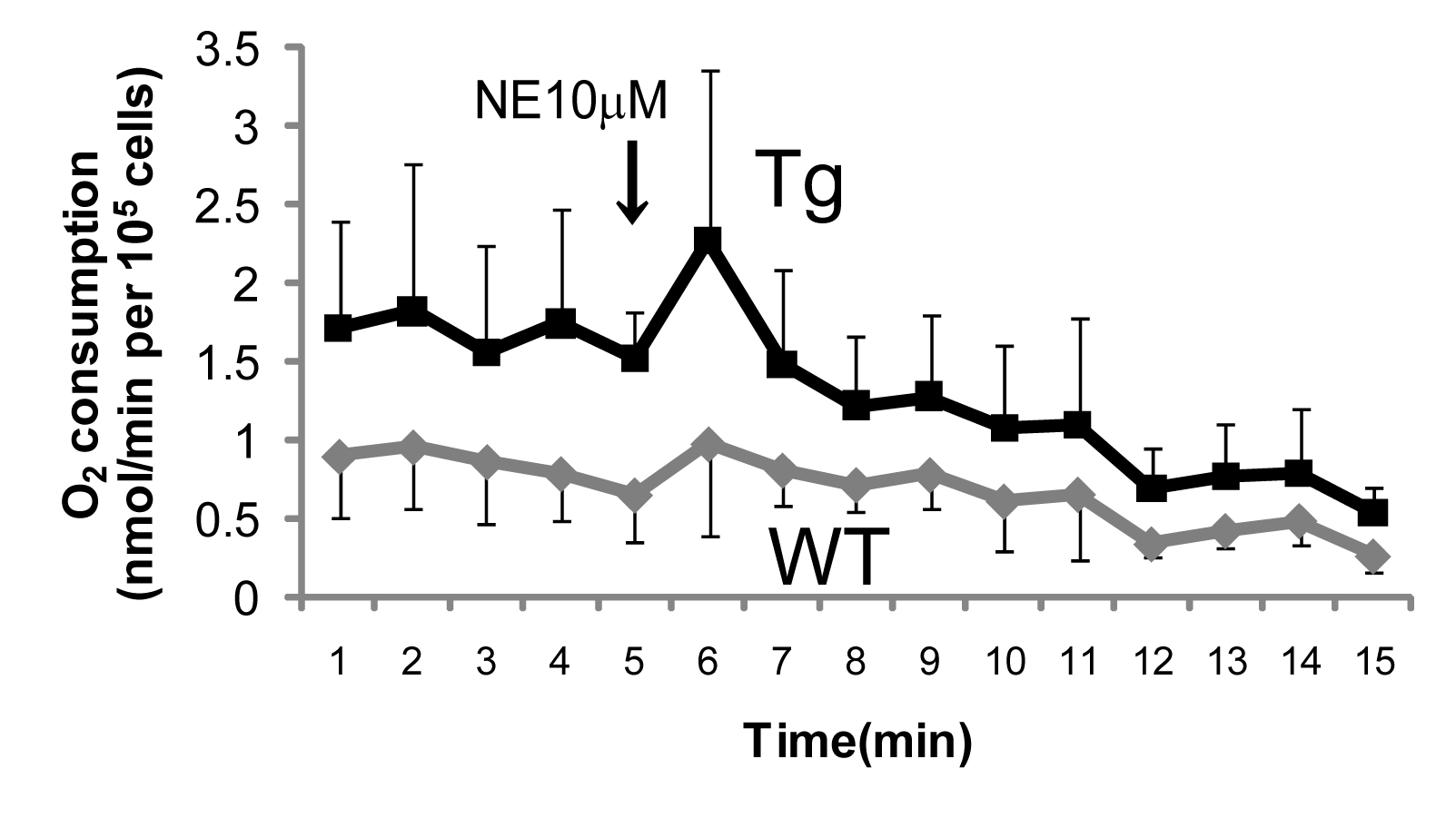

Supplement: Figure 1 — Oxygen consumption of adipocytes isolated from inguinal WAT of wild-type and PeriA Tg mice. An arrow indicates the addition of 10μM norepinephrine (NE). Data are mean ± SEM of values from three independent experiments. (177KB TIF) [file pone.0014006.s001.tif]

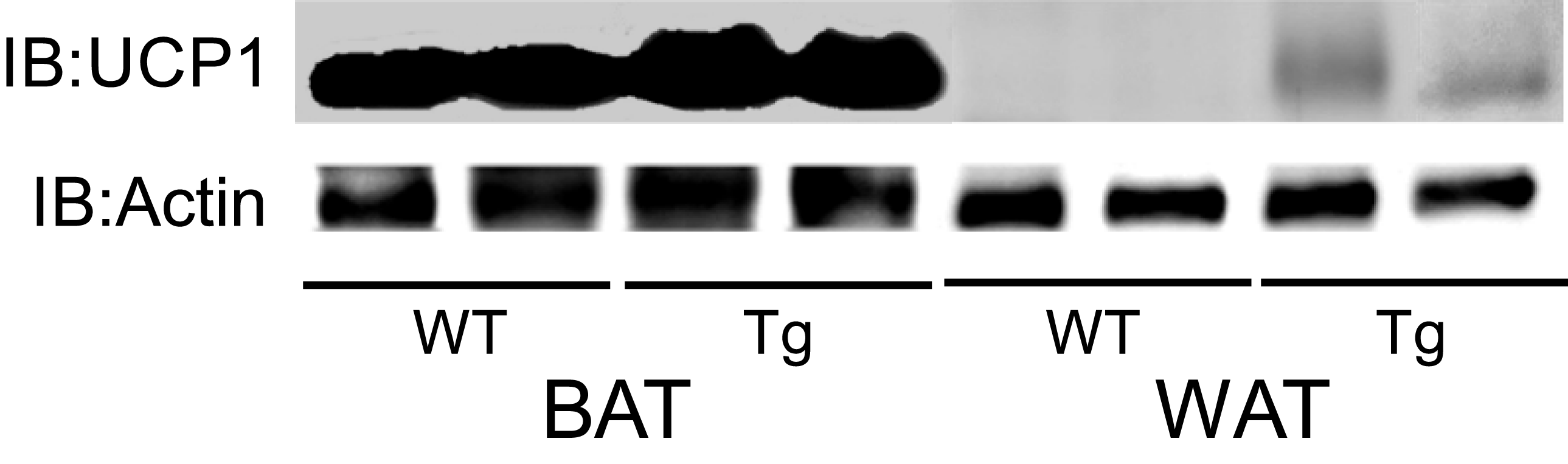

Supplement: Figure 2 — Western blot analysis showing UCP1 protein expression in both BAT and inguinal WAT of WT and Tg mice fed a chow diet. Equal amount (30μg) of proteins was electrophoresed. Actin was used as a loading control. (511KB TIF) [file pone.0014006.s002.tif]
